# Dietary nucleotides supplementation enhances the growth and immune responses of the giant freshwater prawn, *Macrobrachium rosenbergii* (De Man, 1879)

**DOI:** 10.5455/javar.2025.l932

**Published:** 2025-06-25

**Authors:** Belal Hossen, Rokonuzzaman Kibria, Sakib Tahmid Rishan, Muhammad Tofazzal Hossain, Samsul Alam

**Affiliations:** 1 Department of Fisheries Biology and Genetics, Bangladesh Agricultural University, Mymensingh, Bangladesh; 2 Department of Microbiology and Hygiene, Bangladesh Agricultural University, Mymensingh, Bangladesh

**Keywords:** Nucleotide, *Macrobrachium rosenbergii*, growth, hemolymph, immunity, *Aeromonas veronii*

## Abstract

**Objective:**

The study aimed to assess the impact of dietary nucleotide (NT) supplementation on the growth performance and immune response of juvenile *Macrobrachium rosenbergii*.

**Materials and Methods:**

A commercial diet was supplemented with 0.0% (control), 0.10% (T1), 0.15% (T2), and 0.20% (T3) NT for the study. A total of 132 juvenile prawns (0.78 ± 0.03 gm) were randomly distributed into four groups, each having three replicates. Following the 75-day feeding trial, the experimental prawns were exposed to *Aeromonas veronii* at 8.35 × 10^5^ colony forming unit (cfu)/ml for 7 days through a bath treatment.

**Results:**

The growth parameters and survival rate were significantly higher (*p* < 0.05) in NT-supplemented prawns. Immune-related parameters, e.g., total hemocyte count (THC), hemolymph protein, albumin, globulin, superoxide dismutase, and catalase activity, were also significantly higher (*p* < 0.05) in NT-supplemented prawns. The challenge of prawn with *A*. *veronii* resulted in a significant reduction (*p* < 0.05) in THC and other biochemical parameters of hemolymph and caused mortality in all the experimental groups. However, significantly higher survival (*p* < 0.05) against *the A*. *veronii* challenge was found in NT-supplemented prawns.

**Conclusion:**

It can be concluded that dietary NTs should be supplemented at 0.15%–0.2% to enhance the growth, immunity, and resistance of juvenile *M*. *rosenbergii* against *A*. *veronii*.

## Introduction

The farming of the giant freshwater prawn, *Macrobrachium rosenbergii* (De Man, 1879), has been expanded rapidly in Bangladesh due to its suitability for polyculture with other fish species and integrated farming with paddy [1], favorable climatic conditions, rapid growth rate, and wide range of salinity and temperature tolerance [2]. Moreover, it has a very high demand in domestic and international markets due to its nutrient content and delicious taste. According to the Department of Fisheries [3], Bangladesh has been producing an average of 48835.66 metric tons of *M*. *rosenbergii* and exporting 6,079 metric tons of *M*. *rosenbergii* annually since 2012. However, the annual production and export volume of *M. rosenbergii* in Bangladesh has not increased as per potential due to early mass mortality of larvae, post-larvae, and juveniles. The invasion of various pathogens is believed to be the primary cause of the early mass mortality of this valuable species [4–6].

*Aeromonas veronii* is a gram-negative, rod-shaped, facultative anaerobic bacterium widely distributed in aquatic environments and infects various aquatic animals [5]. *A*. *veronii* were found to cause ulcer syndrome in freshwater fish [8] and massive mortality in freshwater prawn, M. rosenbergii [5,7], and yellow catfish, Pelteobagrus fulvidraco [9].

Nucleotides (NTs) are low molecular weight intracellular compounds that play key roles in biochemical processes and are conditionally necessary in the presence of certain physiological challenges, such as growth and development, injury healing, infection, and specific disease states [10]. NTs are found to improve growth and gut health and enhance the resistance of fish and shrimp against bacterial, viral, and parasitic infections via modulating innate and adaptive immune responses [11,12]. Currently, NTs are regarded as semi-essential nutrients [13] and promising immunostimulants that are widely used in aquaculture for sustainable health management of fish and shrimp [11,14,15]. Because of immune-stimulating effects, NTs are regarded as an alternative to antibiotics in the swine industry [16]. The impact of dietary NTs has been assessed on the growth performance, gut health, immune response, and disease resistance of red seabream, *Pagrus major* [17]; red drum, *Sciaenops* [18]; Asian sea bass, *Lates calcarifer* [14]; European sea bass, *Dicentrarchus labrax* L [19]; gilthead seabream, *Sparus aurata* [20]; giant freshwater prawn, *M*. *rosenbergii* [21]; and Pacific white shrimp, *Litopenaeus vannamei* [15,22–26]. However, to solve the existing early mortality problem of prawn culture in Bangladesh, the effects of dietary NT supplements on the M. rosenbergii population of Bangladesh have not been reported yet. The present study was undertaken to determine the effects of dietary NTs on the growth performance, immune response, and resistance of M. rosenbergii against A. veronii as a preliminary attempt to solve the early mortality problem that badly hampers the prawn culture in Bangladesh.

## Materials and Methods

### Ethical approval

The approval of the research work and use of prawns as research materials was done by the Institutional Ethical Committee, Bangladesh Agricultural University (Approval No.: ESRC/FISH/41).

### Experimental design

**Figure 1.**
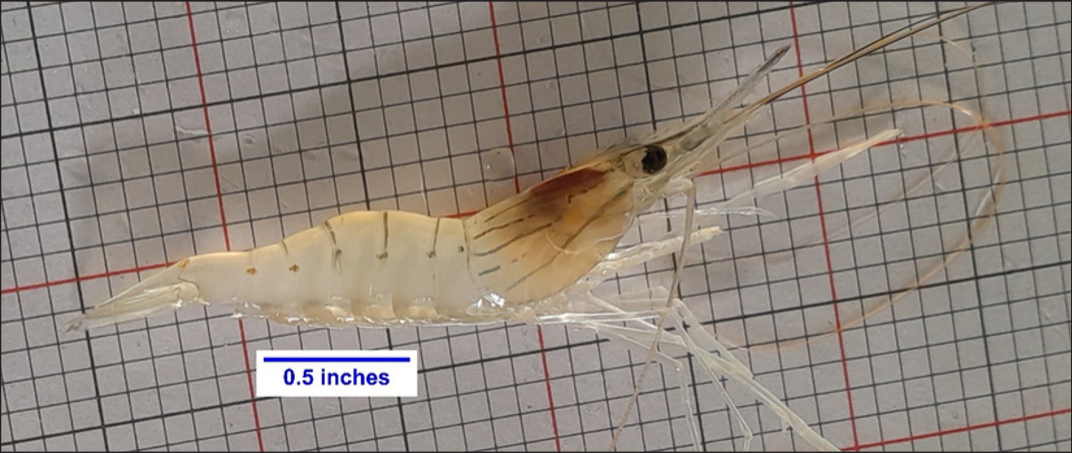
juvenile of *M. rosenberrgii*, used in the experiment.

A total of 132 healthy and robust juvenile *M. rosenbergii* (Fig. 1) (0.78 ± 0.03 gm) were collected from a nursery pond and acclimatized in the aquarium for 7 days before starting the experiment. Twelve glass aquaria (50 × 30 × 30 cm) filled with 30 L of water equipped with an aerator (Resun, ACO-004, China) were divided randomly into four experimental groups, namely, Control (commercial diet with no NT), T1 (0.1% NT), T2 (0.15% NT), and T3 (0.2% NT), with three replications for each following a completely randomized design. Each aquarium was covered by a nylon net to avoid the escape of the prawn from the aquarium. PVC pipes (6 inches long and 0.75 inches in diameter) were used as artificial shelters to avoid cannibalism during the molting phase (Fig. 1).

### Experimental diet preparation

The commercial prawn feed, CP shrimp/prawn nursery feed, is manufactured and marketed by C.P. Aquaculture (India) Pvt. Ltd. (Ingredients: fish meal, soybean meal, wheat flour, raw rice bran, fish oil, phospholipids, lecithin, vitamins, and minerals premix; Proximate composition: moisture 12.33, protein 37.17, lipid 6.23, ash 14.23, crude fiber 4.25, and carbohydrate 25.79) was used as control feed in this experiment. A purified mixture of NT, a balanced mixture of inosine monophosphate, adenosine monophosphate, guanosine monophosphate, uridine monophosphate, and cytidine monophosphate at a ratio of 1:1:1:1:1 (Nucleotide Nutrition Ltd., Switzerland), was homogeneously mixed with commercial prawn feed by spraying at four different concentrations, e.g., 0.0% (control), 0.10% (T1), 0.15% (T2), and 0.20% (T3). A total of 450 mg commercial NT powder was mixed thoroughly into 9 ml distilled water (DW) and used as a stock solution (1 ml solution is equivalent to 50 mg NT). Then, to mix the NT homogenously, 2 ml of NT stock solution was diluted by 6 ml of DW (total 8 ml) and sprayed over 100 gm of feed for T1 (0.1% NT). Similarly, 3 ml of NT stock solution was diluted by 5 ml of DW (total 8 ml) for T2 (0.15% NT), and 4 ml of stock solution was diluted by 4 ml of DW (total 8 ml) for T3 (0.2% NT), and then sprayed over the same amount of feed. Besides, 100 gm of control feed was taken in which no NT was added, but only 8 ml of DW was sprayed over the feed. Then, a commercial gel (Growth gel, brand: Advanced Chemical Industries, Dhaka, Bangladesh) was used as a binder to coat the NT with the feed. Finally, the feed was air-dried for about an hour and then stored at 4°C in four tightly sealed plastic bottles.

### Feeding and water quality monitoring

The prawns were reared for 75 days with respective experimental diets. The feed was supplied at 10% of the body weight twice a day at 8:00 h and 18:00 h. The tanks were siphoned regularly to clean unused feed and metabolic wastes. Half of the water in the aquarium was replaced at one-day intervals. Continuous aeration was done by an air pump (Resun, ACO-004, China). Water quality parameters were checked and recorded routinely from the start to the end of the experimental period to ensure a suitable range of water parameters. Temperature, pH, and total dissolved solids (TDSs) were measured by a digital water quality tester (Hanna Instruments Inc., Romania, www.hannainst. com). Dissolved oxygen (DO) was measured by a DO meter (Lutron Model 5509, Taiwan), and the ammonia level was checked by an Ammonia NH_3_ /NH^+^_4_ test kit (Mars Fishcare North America, Inc., USA). The important water quality parameters, e.g., temperature, pH, DO, TDS, and ammonia, measured from the experimental pond throughout the experimental period (Table 1) were found within the acceptable range for freshwater prawn culture [27].

**Table 1.** Different water quality parameters (mean ± SD) of the experimental group during the experimental period.

Water parameter	Control	T_1_ (0.10% NT)	T_2_ (0.15% NT)	T_3_ (0.20% NT)	*p*-value
Temperature (°C)	28.27 ± 1.32	28.93 ± 1.33	28.83 ± 1.24	28.91 ± 1.33	0.115
pH	7.86 ± 0.26	7.85 ± 0.23	7.81 ± 0.28	7.87 ± 0.33	0.822
TDS (ppm)	148.30 ± 5.66	147.00 ± 6.61	149.64 ± 6.27	148.28 ± 4.88	0.324
DO (ppm)	5.99 ± 0.47	5.89 ± 0.42	5.91 ± 0.31	5.93 ± 0.39	0.725
Alkalinity (ppm)	117.67 ± 5.83	119.44 ± 4.91	118.64 ± 5.21	120.25 ± 4.52	0.191
Ammonia (ppm)	0.72 ± 0.11	0.69 ± 0.09	0.71 ± 0.10	0.74 ± 0.10	0.208

Values are expressed as mean ± SD.

• Weight gain (WG) (%) = {(Final weight - Initial weight)/Initial weight} × 100
• Specific Growth Rate (SGR) = {(ln Final weight - ln Initial weight)/days} × 100
• Feed Conversion Ratio (FCR) = Dry feed consumed (gm)/Live WG (gm)
• Protein Efficiency Ratio (PER) = Live WG (gm)/Dry protein intake (gm)
• Survival (%) = (Total number harvested/Total number stocked) × 100

### Observation of immune-related parameters

On completion of the feeding trial, six prawns were taken from each of the treatments (two from each of the tanks) for hemolymph collection. Hemolymph (100 μl) was drawn into a microcentrifuge tube (1.5 ml) from the pleopod base of the second abdominal segment using a sterile 1 cc syringe (25 G × 13 mm needle) loaded with 300 μl pre-cooled (4°C) 10 mM ethylene diamine tetra acetic acid solution to avoid coagulation of hemolymph. The hemolymph and the anticoagulant solution were mixed homogeneously and stored on ice. This hemolymph was used to count total hemocytes and estimate the levels of protein, albumin, globulin, superoxide dismutase (SOD), and catalase (CAT) activity.

### Total hemocyte count (THC)

To count the total hemocyte, 10 μl of 1% Trypan Blue dye was mixed with 100 μl hemolymph and then stored on ice for 20 min for staining. Then, 5 μl of stained hemolymph was placed on a hemocytometer (Neubauer) and observed under an optical microscope (Olympus-CX21, Japan) connected to a desktop computer. Values were expressed as million hemocytes/ml.

### Observation of growth parameters and survival rates (SRs)

At the end of the feeding trial, all the prawns were weighed separately, and the growth and SRs were estimated using the following equations:

### Determination of total hemolymph protein, albumin, and globulin

Total hemolymph protein was measured using a commercial kit (Total protein, Chemelex, S.A., Pol. Ind. Can Castells-C/Industria 113 Nave J, 08420 Canovelles, Barcelona, Spain) following the manufacturer’s instructions. Similarly, albumin concentration was measured using a commercial kit (Albumin, Linear Chemicals S.L.U, Barcelona, Spain) following the manufacturer’s instructions. A semi-automatic clinical chemistry analyzer (SA-20 CLINDIAG) was used to measure the absorbance at a wavelength of 540 and 630 nm for protein and albumin, respectively. Globulin was calculated after subtracting the albumin content from the total protein [28].

### Determination of SOD and CAT activity

The SOD activity of hemolymph was measured with a commercial kit (SOD Assay Kit, Beijing Solarbio Science and Technology Co., Ltd.) by following the manufacturer’s instructions. Similarly, CAT activity was measured with a commercial kit (CAT Activity Assay Kit, Beijing Solarbio Science and Technology Co., Ltd.) by following the manufacturer’s instructions. An Eppendorf Bio-Spectrophotometer was used to measure the absorbance at a wavelength of 560 and 240 nm for SOD and CAT, respectively.

### Bacterial challenge with Aeromonas veronii

After the feeding trial, the experimental prawns were challenged with a virulent strain of A. veronii collected from the laboratory repository of the Department of Microbiology and Hygiene, Bangladesh Agricultural University, Mymensingh, previously isolated and characterized by [29] from the liver of a diseased (ulcerative lesions) stinging catfish, *Heteropneustes fossilis*. The pathogenicity of the *A. veronii* in *M. rosenbergii* was reported by [5,7]. 10 ml of bacterial solution containing 8.35 × 105 cfu/ml was mixed with aquarium water. The dose of bacterial solution was determined based on the findings of [5]. Eighteen prawns from each of the experimental groups (six from each tank) were subjected to bacterial challenge for 7 days, maintaining three replications for each of the experimental groups. During the challenge period, the prawns were fed twice per day at 5% of their body weight with a NT-free control diet. The mortality was checked regularly for seven days, and the cumulative mortality was calculated. The immune-related parameters of the hemolymph of the surviving prawns were estimated after the completion of the challenge test.

### Statistical analysis

The Shapiro–Wilk test was done to check the distribution of the data. The statistical significance of the differences was calculated among the control and the treated groups via one-way analysis of variance (ANOVA) and the TUKEY test using Statistical package for social science (SPSS) (ver. 22). Besides, a paired sample t-test was done to compare the immune-related parameters before and after the *A*. *veronii* challenge. All the values were reported as mean ± standard error, and the differences between experimental groups were considered significant at *p* ≤ 0.05. Microsoft Excel (ver. 2010) and SPSS (ver. 25, IBM Corp., New York, USA) were used to process and analyze the data.

## Results

### Effect of NT on growth, feed utilization, and survival of M. rosenbergii

The mean initial weight of the prawns stocked in the different experimental groups was almost the same (*p* > 0.05). At the end of the feeding trial, significantly higher (*p* < 0.05) growth in terms of final body weight (FBW), WG, and SGR was found in prawns fed a NT-enriched diet (Table 2)*.* Besides, a significantly lower (*p* < 0.05) FCR and significantly higher (*p* < 0.05) PER were found in NT-supplemented groups than those of the control group (Table 2).

Supplementation of NT also positively affected the survival of *M*. *rosenbergii*. Maximum survival was found in prawns fed a 0.15% NT (T1) supplemented diet, which was significantly higher than the control group (Table 2).

**Table 2.** Growth performance and feed utilization of prawns fed nucleotide-supplemented diets.

Parameters	C (0.0% NT)	T_1_ (0.10% NT)	T_2_ (0.15% NT)	T_3_ (0.20% NT)	*p*-value
IBW (gm)	0.77 ± 0.05	0.78 ± 0.06	0.77 ± 0.07	0.79 ± 0.05	0.993
FBW (gm)	4.23 ± 0.07^c^	4.91 ± 0.08^b^	5.21 ± 0.09^b^	5.56 ± 0.11^a^	0.000
WG (%)	456.42 ± 32.67^b^	628.49 ± 68.54^ab^	718.56 ± 81.49^a^	617.93 ± 44.54^ab^	0.041
SGR (%)	2.23 ± 0.08^b^	2.51 ± 0.11^ab^	2.64 ± 0.12^a^	2.56 ± 0.08^ab^	0.041
FCR	1.87 ± 0.14^a^	1.57 ± 0.12^ab^	1.41 ± 0.13^b^	1.39 ± 0.08^b^	0.029
PER	1.64 ± 0.12^b^	2.20 ± 0.25^ab^	2.57 ±0.28^a^	2.17 ± 0.16^ab^	0.045
SR (%)	72.73 ± 5.25^b^	87.88 ± 3.03^ab^	90.91 ± 0.00^a^	87.88 ± 3.03^ab^	0.020

Values are means ± SE of three replicates. Mean values in the same rows with different superscripts indicate significant differences (*p* < 0.05) among the experimental groups.

FBW, final body weight; FCR, feed conversion ratio; IBW, initial body weight; PER, protein efficiency ratio; SGR, specific growth rate; SR, survival; WG, weight gain.

### Effects of NTs on THCs

**Figure 2.**
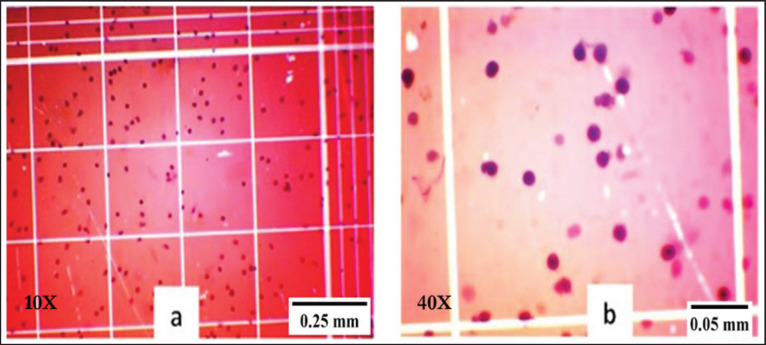
Trypan blue stained hemocytes on Nuber’s hemocytometer under a microscope. (a) 10x and (b) 40x magnification.

The THC (Fig. 2) was checked at two phases—before and after the bacterial challenge test. During the first phase, the maximum hemocyte count (5.20 ± 0.08 million/ml) was found in T2 (0.20% NT), which was significantly higher (*p* < 0.05) than that of the control (C) and T1 (0.10% NT) groups (Table 3). A similar trend was also found during the second phase of the hemocyte count. Besides, we observed a significantly reduced (*p* < 0.05, paired sample *t*-test) number of hemocytes in all the experimental groups due to the infection of *A*. *veronii* (Table 3).

**Table 3.** THC of prawns of different experimental groups before and after the bacterial challenge.

Experimental group	Pre-challenge THC (million/ml)	Post-challenge THC (million/ml)	t-test	*p*-value
Control (0% NT)	3.55 ± 0.16c	3.21 ± 0.12c	3.306	0.021*
T1 (0.10% NT)	4.19 ± 0.09^b^	4.04 ± 0.06^b^	3.124	0.026*
T2 (0.15% NT)	4.89 ± 0.06^a^	4.39 ± 0.11^ab^	4.039	0.010*
T3 (0.20% NT)	5.20 ± 0.08^a^	4.65 ± 0.07^a^	3.887	0.012*
F value	49.32	46.68	-	-
p value	< 0.001	< 0.001	-	-

Values are means ± SEM of three replicates. Mean values in the same column with the different superscript letters differ significantly (*p* < 0.05, one-way ANOVA).

* Indicates a significant difference in the case of t-test.

### Effects of NTs on hemolymph protein, albumin, and globulin levels of M. rosenbergii

The hemolymph proteins, albumin, and globulin, were checked at phase, before and after the bacterial challenge, and shown in Table 4. The maximum level of hemolymph protein, albumin, and globulin was found in T3 (0.2% NT) and the minimum in the control (0.0% NT). The hemolymph proteins albumin and globulin in NT-supplemented prawns were significantly higher (*p* < 0.05) than in the control groups before the bacterial challenge (just after the feeding trial) as well as after the bacterial challenge. We observed a reduced level of hemolymph protein, albumin, and globulin in all the groups of the experimental prawn after the bacterial challenge. However, a significant reduction (*p* < 0.05) in all the parameters was found in the control group.

### Effects of NTs on SOD and CAT activity

On completion (75 days) of the feeding trial, we observed that the hemolymph of the prawns fed a NT-enriched diet showed significantly higher (*p* < 0.05) levels of SOD and CAT activity than those of the control group (Table 5). Similarly, after the bacterial challenge *A*. *veronii,* the SOD and CAT enzyme activity was also found significantly higher (*p* < 0.05) in NT-supplemented prawns than in those of the control group prawns. A reduced level of SOD and CAT enzyme activity in hemolymph was found after the 7-days bacterial challenge with *A*. *veronii* (Table 5). The reduction level of both the SOD and CAT was statistically significant (*p* < 0.05, paired sample *t*-test) in control and T1 (Table 5).

**Table 5.** Level of SOD and CAT activity in the hemolymph of the prawn fed with different levels of nucleotide-supplemented diets.

	Experimental group	Before bacterial challenge	After bacterial challenge	*t*-value	*p*-value
	Control (0.0% NT)	109.52 ± 1.07^c^	105.19 ± 1.11^d^	5.16	0.004*
	T1 (0.10% NT)	134.42 ± 2.07^b^	125.66 ± 1.13^c^	4.19	0.009*
SOD (U/ml)	T2 (0.15% NT)	139.64 ± 1.08^b^	136.31 ± 1.45^b^	2.32	0.068
	T3 (0.20% NT)	147.57 ± 2.34^a^	144.34 ± 1.92^a^	2.15	0.084
	F-value	71.59	138.71	-	-
	p-value	<0.001	<0.001	-	-
	Control (0.0% NT)	65.95 ± 0.92^c^	62.93 ± 0.97^d^	5.15	0.004*
	T1 (0.10% NT)	72.61 ± 1.38^b^	69.02 ± 0.55^c^	2.81	0.038*
CAT (U/ml)	T2 (0.15% NT)	79.26 ± 1.5^a^	76.42 ± 1.04^b^	2.35	0.066
	T3 (0.20% NT)	84.37 ± 2.31^a^	81.00 ± 0.37^a^	1.66	0.158
	F-value	24.74	103.36	-	-
	p-value	<0.001	<0.001	-	-

Values are means ± SEM of three replicates (*n* = 24, 6 for each experimental group). Mean values in the same column with the different superscript letters differ significantly (*p* < 0.05). * Indicates a significant difference (*p* < 0.05, paired sample *t*-test) in a particular experimental group pre and post bacterial challenge with *A*. *veronii*.

### Effects of NTs on SRs of the prawns during the bacterial challenge test

**Figure 3.**
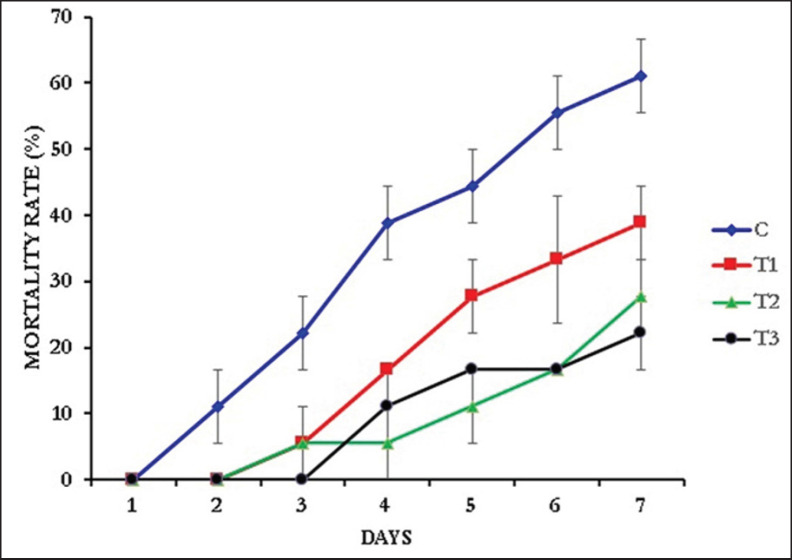
The cumulative morality pattern observed during the challenge test with *A. veronii*.

**Figure 4.**
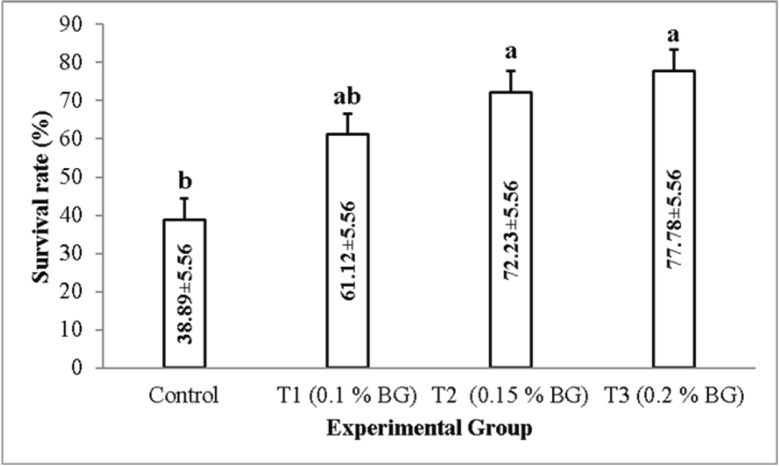
Survival rate (%) at the end the challenge test with *A. veronii.* Different letters on the bar indicates significant differrences among the experiental group.

Prawns of the control group were found to be more susceptible to the *A*. *veronii,* as the mortality started in the prawns of the control group just 1 day after the bacterial exposure. On the other hand, the mortality in T1 (0.1% NT) and T2 (0.15% NT) started after 2 days, while in T3 (0.2% NT), the mortality started after 3 days of bacterial exposure (Fig. 3). At the end of the 7-day bacterial challenge with *A*. *veronii* (8.35×105 cfu/ml), maximum survival (77.78 ± 5.56) was found in T3 (0.2% NT) and minimum (38.89 ± 5.56) in the control (0.0% NT). The SR of the NT-supplemented prawns against *A*. *veronii* was significantly higher (one-way ANOVA, F = 9.583, *p* = 0.005) than the control group (Fig. 4).

## Discussion

The present study investigated the effects of dietary NT on growth, immune response, and resistance of *M*. *rosenbergii* against *A*. *veronii* infection. The dose of NT was built upon [21], but the way of incorporation of the NTs into the experimental diet differed from [21]. We sprayed the suspension of NT directly over the commercially formulated feed at respective doses and coated the NT with the feed using a food-grade gel to avoid leaching. The simplicity of the method we followed in the present study to incorporate NTs with the commercial diet will be easy to repeat for other researchers as well as the marginal prawn farmers.

### Effects of NTs on growth, feed utilization, and survival of M. rosenbergii

Usually, the systematic growth of living animals occurs through the de novo synthesis of purines and pyrimidines [30]. However, during the early growth phase of animals [11] as well as in stressed situations [24], extrinsic provision of dietary NTs is an effective way besides the *de novo* synthesis of NTs to support the rapid cell division during the early stage of life and thus enhance the growth of fish and crustacean shellfish [11,13,19,24]. In the present study, the NT-enriched diet was found to significantly enhance the growth and survival of *M*. *rosenbergii* (Table 2), which indicates a positive impact of NT supplementation. Besides, the significantly higher PER and significantly lower FCR (Table 2) of the NT-enriched food in the current study indicated that the experimental prawn used the NT-containing meal more effectively than the control feed. The findings of the study regarding growth, feed utilization, and survival are supported by the results previously reported by [21–23,31].

However, some authors did not find any positive effects of NT supplementation on the growth and survival of the Pacific white shrimp, *L*. *vannamei* [24], and red drum, *Sciaenops ocellatus* [18]. The dose and type of NTs, water parameter of the experimental unit, physiological condition of the experimental animal, duration of feeding trial, and so on, may also result in variation in growth performance.

### Effects of NTs on hemocyte counts

In the absence of an adaptive (acquired) immune system, crustaceans usually defend against any type of pathogenic attack using their innate (nonspecific) immune system [32]. According to [33], hemocytes play a vital role in the immunity of crustaceans and other invertebrates through phagocytosis, encapsulation, and lysis of invading microorganisms. The number of hemocytes in different crustacean shellfish becomes enhanced due to dietary supplementation of immunostimulants like NT [21,25,26], β-glucan [34], glycyrrhizin [35], and mannan oligosaccharide [23], and the number of hemocytes becomes reduced rapidly in times of pathogenic infections [36–38]. The present study reported significantly higher THC in *M*. *rosenbergii* fed with NT-supplemented diets (Table 3), which is supported by the findings reported by [21–23].

Besides, a significant reduction in hemocytes was observed in all the experimental groups after the bacterial challenge with *A*. *veronii* (Table 3). The reduction in THC could happen due to the attack of the *A*. *veronii.* Previously, the decline of THC was reported in *Litopenaeus vannamei* due to the infection with *Vibrio* spp. [37] and Taura syndrome virus [38].

### Effects of NTs on hemolymph protein, albumin, and globulin

According to [39], proteins in the hemolymph serve a vital role in crustaceans’ life cycles, ranging from oxygen delivery to reproduction to regulating the animal’s responses to external stimuli. The amounts of serum protein and globulin indirectly indicate the particular humoral immunological status [40], and the total protein, albumin, and globulin concentrations in the blood of fish and shellfish may alter due to the supplementation of various immunostimulants. For example, [41] reported significantly enhanced serum protein, albumin, and globulin in *Cyprinus carpio* fed with a levamisole-supplemented diet; [42] reported significantly enhanced total protein and albumin in dietary piperine-supplemented *L*. *vannamei*; and [34] observed a significantly higher level of hemolymph protein in β-glucan-supplemented *M*. *rosenbergii*. Similarly, in the present investigation, we also found significantly higher levels of hemolymph protein, albumin, and globulin in NT-supplemented prawns, indicating better health with improved immunity. Because of the bacterial challenge with *A*. *veronii*, a significant reduction of hemolymph protein, albumin, and globulin in the control group indicates the NT-supplemented prawns showed improved immunity and resistance against *A*. *veronii* (Table 4). The decline of hemolymph protein, albumin, and globulin because of the challenge with *A*. *veronii* was found to be consistent with [41], who observed reduced levels of serum protein, albumin, and globulin in *Cyprinus carpio* due to infection with the *A*. *hydrophila*.

**Table 4.** Level of hemolymph protein, albumin and globulin among the experimental groups.

Parameter	Experimental group	Before challenge	After challenge	*t*-test	*p*-value
	Control	7.06 ± 0.09^c^	6.46 ± 0.24^b^	3.11	0.027*
Hemolymph protein (gm/dl)	T1 (0.10% NT)	7.88 ± 0.17^b^	7.19 ± 0.34^ab^	2.24	0.076
	T2 (0.15% NT)	8.17 ± 0.02^ab^	7.86 ± 0.16^a^	2.25	0.075
	T3 (0.20% NT)	8.35 ± 0.03^a^	8.13 ± 0.32^a^	0.70	0.516
	F	34.19	7.42	-	-
	p	0.000	0.002		
	Control	0.63 ± 0.01^c^	0.55 ± 0.01^d^	4.49	0.006*
Hemolymph Albumin (gm/dl)	T1 (0.10% NT)	0.71 ± 0.02^b^	0.63 ± 0.01^c^	3.19	0.024*
	T2 (0.15% NT)	0.75 ± 0.02^b^	0.72 ± 0.01^b^	1.51	0.191
	T3 (0.20% NT)	0.85 ± 0.19^a^	0.83 ± 0.01^a^	1.87	0.120
	F	26.16	87.20	-	-
	p	0.000	0.000	-	-
	Control	6.44 ± 0.09^b^	6.91 ± 0.24^b^	2.86	0.03*
Hemolymph Globulin (gm/dl)	T1 (0.10% NT)	7.18 ± 0.17^a^	6.57 ± 0.34^ab^	1.92	0.11
	T2 (0.15% NT)	7.43 ± 0.03^a^	7.15 ± 0.17^a^	1.85	0.12
	T3 (0.20% NT)	7.50 ± 0.02^a^	7.30 ± 0.32^a^	0.63	0.56
	F	24.02	5.31	-	-
	p	0.000	0.007	-	-

Values represent the means ± S.E. of three replicates (*n* = 24, 6 for each experimental group). Values in the same column with different superscript letters indicate significant differences among the experimental group (*p* < 0.05, one-way ANOVA). *Indicates a significant difference (*p* < 0.05, paired sample t-test) in a particular experimental group pre and post bacterial challenge with *A*. *veronii*.

### Effects of NTs on SOD and CAT activity

SOD and CAT are two important antioxidant enzymes broadly found in plants, animals, microorganisms, and cultured cells. Both of these enzymes are regarded as potential indicators of oxidative stress for aquatic animals [43] as they protect the cells from oxidative damage. SOD, the main H₂O₂-producing enzyme, catalyzes the dismutation of superoxide anion (O₂⁻) to form H₂O₂ and O₂ and thus scavenges superoxide anion from the tissue [44,45]. On the other hand, CAT, the main H₂O₂ clearing enzyme, breaks down hydrogen peroxide (H₂O₂) to produce water and oxygen and thus prevents oxidative damage and plays a vital role in the detoxification process [46,47]. One unit of CAT is defined as the amount of enzyme needed to reduce 1 μmol of H₂O₂/min [48], and one unit of SOD activity is defined as the amount required to inhibit the rate of xanthine reduction by 50% in a 1 ml reaction system [45] and expressed as a unit/ml. The present study showed significant improvement in the SOD and CAT activity in the hemolymph of the NT-supplemented *M*. *rosenbergii*, indicating a positive effect of dietary NTs on the immunity of *M*. *rosenbergii*. Similarly, significant improvement of SOD and CAT antioxidants was also reported in sterlet sturgeon, *Acipenser ruthenus* [48], and *M*. *rosenbergii* [24] due to NT supplementation. Conversely, [17] observed a significant reduction in serum CAT level in NT-supplemented red sea bream, *Pagrus major*. However, the SOD and CAT levels in several fish and crustacean species were also found to be affected by some other feed supplements like β-glucan [34, 47, 49], β-carotene [47], dietary arginine [44], threonine [45], and so on. We found a significant reduction in SOD and CAT activity in the control and T1 (0.1% NT) due to the challenge with *A*. *veronii* (Table 5), which was supported by Ardiansyah et al. [50], who observed a significant decline in SOD and CAT activity in Pacific white shrimp, *Litopenaeus vannamei,* 72 h post infection with white feces syndrome (WFS) virus. The activity of these two antioxidant enzymes in serum or hemolymph is a positive indicator of improved immunity.

### Effects of NTs on resistance against Aeromonas veronii

*Aeromonas veronii* is a pathogenic bacterium for fish [8,9,51] and crustaceans [5,7]. According to Li et al. [11], post-challenge survival of experimental animals with particular pathogens is usually evaluated as a measure of disease resistance. Although the direct antimicrobial effects of the dietary NTs are practically unknown, similar to many other immunostimulants, exogenous supply of dietary NTs is reported to resist the pathogenic attack through the modulation of immune-related parameters of the host [12]. Previously, Chen et al. [31] reported enhanced resistance in *M*. *rosenbergii* against *Vibrio anguillarum,* and Andrino et al. [23], Guo et al. [24], Segarra et al. [26] and Novriadi et al. [15], Novriadi et al. [25] reported enhanced resistance in *L*. *vannamei* against white spot syndrome virus, *Vibrio parahaemolyticus,* and *Vibrio harveyi*, respectively, due to the supplementation of NTs. Similar to the previously mentioned study, the current study also revealed significantly higher survival of the prawns against *A*. *veronii* in NT-supplemented groups, indicating that the antibacterial ability of the experimental *M*. *rosenbergii* against *A*. *veronii* became enhanced due to the NT supplementation.

## Conclusion

The present study showed that the dietary NT supplementation at the dose of 0.15%–0.2% was beneficial for improving growth performance, immune response, and resistance of juvenile *M*. *rosenbergii* to *A*. *veronii.* The findings of the present study might be beneficial to minimizing the early mortality problem that is currently facing the prawn sector of Bangladesh. Although the present study positively evaluated the effects of dietary NT on the growth, immune response, and resistance of juvenile *M*. *rosenbergii* against pathogenic *A*. *veronii* in indoor aquarium conditions, it could not determine whether the dietary NT would similarly affect the growth and immunity of the larvae, post-larvae, and adult stages of *M*. *rosenbergii.* Moreover, the function of the dietary NT in comparatively larger outdoor conditions, like a pond ecosystem, needs to be addressed before the large-scale application of the dietary NT in the commercial aquaculture sector.

## List of abbreviations

ANOVA, analysis of variance

CAT, catalase

cfu, colony forming unit

DO, dissolved oxygen

DW, distilled water

FBW, final body weight

FCR, feed conversion ratio

NT, nucleotide

PER, protein efficiency ratio

SGR, specific growth rate

SOD, superoxide dismutase

SPSS, Statistical package for social science

SR, survival rate

THC, total hemocyte count

TDS, total dissolved solids.
